# EcoScale, a semi-quantitative tool to select an organic preparation based on economical and ecological parameters

**DOI:** 10.1186/1860-5397-2-3

**Published:** 2006-03-03

**Authors:** Koen Van Aken, Lucjan Strekowski, Luc Patiny

**Affiliations:** 1 EcoSynth, Stationstraat 123, B-8400 Oostende, Belgium; 2 Georgia State University, Department of Chemistry, University Plaza Atlanta, Georgia 30302-4098, USA; 3 Federal Institute of Technology Lausanne, Institute of Chemical Sciences and Engineering, EPFL-ISIC, CH-1015 Lausanne, Switzerland

## Abstract

A novel post-synthesis analysis tool is presented which evaluates quality of the organic preparation based on yield, cost, safety, conditions and ease of workup/purification. The proposed approach is based on assigning a range of penalty points to these parameters. This semi-quantitative analysis can easily be modified by other synthetic chemists who may feel that some parameters should be assigned different relative penalty points. It is a powerful tool to compare several preparations of the same product based on safety, economical and ecological features.

## Introduction

The acceptable preparation of an organic product involves not only a relatively efficient reaction but also the ease of workup and purification. Safety and ecological friendliness are also of paramount importance. Therefore, in order to evaluate the quality of the overall preparation process, it is important to examine all its components.

To address this issue, some partial metrics for the preparation efficiency have been developed. They are mainly used as a predictive tool for chemical processes on a larger scale when substituting a traditional chemical process with an alternative. [1–2] The main parameters and approaches are briefly discussed as follows.

### Atom economy [3–4]

This parameter is the ratio of the molecular weight of the target molecule to the sum total of the molecular weights of all the substances produced in the stoichiometric equation for the reaction involved. It takes into account the amount of the reagents incorporated into the end product. Cycloadditions are examples of transformations with 100% atom economy. For other reactions (e.g. substitution reaction), a 100 % economy can never be reached due to the intrinsic nature of the reaction. The main use of this parameter is to adapt reaction sequences in a way that transformations with low atom economy are limited to a minimum.

### Environmental factor (E-factor) [5–8]

This factor is the ratio of the weight of generated waste to the total weight of the end product. It is a useful tool for rapid evaluation of processes based on generated waste. For example, the comparison of E-factors of the homogeneous and heterogeneous catalytic processes in the alkylation of benzene shows a 30-fold preference towards the heterogeneous method. Recently, it has also been applied to assess the development of an environmentally benign synthesis of sildenafil citrate (Viagra™). [9] The E-factor for the final process is very low with just 6 Kg of waste per kilogram of product compared with an industry average of 25–100 Kg.

### Environmental quotient (EQ) [10]

The value of the E-factor is limited as it does not take into account the nature and environmental impact of the generated waste. In order to arrive at a more meaningful prediction, the E-factor is multiplied by a environmentally hazardous quotient Q. For example, a Q value of 1 can be attributed to NaCl, while heavy metals can be assigned a value between 100–1000 on the basis of their toxicity. Based on the environmental quotient, a computer program has been developed (EATOS of Environmental Assessment Tool for Organic Synthesis) [11] that can be used to compare and improve chemical reactions.

### Effective mass yield [12]

This parameter is defined as the percentage of the mass of desired product relative to the mass of all non-benign materials used in the synthesis. It introduces the important issue of (eco)toxicity.

### Mass intensity [13]

The mass intensity is defined as the ratio of the total mass used in a process (step) and the mass of the end product. It takes into account the yield, stoichiometry, solvent, and the reagents used in synthesis. The total mass also includes chemicals (except water) used in workup procedures such as washes with acid, base, salt solution or organic solvent, as well as extractions and/or crystallizations.

Also, a few unified metrics has been developed which combine some of the above mentioned individual parameters and factors relevant for specific purposes.

### The process profile [14]

Intended primarily as a management tool for economic evaluation, it takes into account all important factors involved in large scale production. These are process parameters, raw material cost, yield, throughput time, throughput volume, number of steps in synthetic sequence, special equipment requirements, reproducility, tolerance to abuse, linearity of sequence, environmental abuse potential, potential occupational health and safety hazards, raw material availability, susceptibility to regulatory changes and patent protection.

### Life cycle analysis (LCA) [15–16]

In this methodology, all stages of the life cycle of a chemical as well as environmental impacts of by-products and auxiliaries (solvents, co-reagents, and technical facilities) are considered. It consists of three domains: the analysis of the starting material, the analysis of the impact, and the analysis of the improvements. It can be used to evaluate existing processes and/or design new processes.

### Proprietary metrics

The above analyses often show that the cost of waste, including effluent treatment, waste disposal, loss of raw materials, etc., can amount up to 40% of the overall production costs. [17] This understanding has led to several governmental (e.g. Green Chemistry Program of the U.S. Environmental Protection Agency [18]) and corporate initiatives to develop their own set of qualitative and semiquantitative green parameters. For example, GlaxoSmithKline has published a set of metrics such as carbon efficiency (CE) and reaction mass efficiency (RME) which enables an assessment to be made of batch processes in terms of waste, energy usage, and chemistry efficiency. [19] These metrics are based on the number of chemistry steps, number of purification steps, number of isolated intermediates, total yield, nature of solvents, the use of extreme conditions, and the use of reagents with known environmental, safety or health problems, among others.

### Unification of reaction metrics for green chemistry

The development of a new reaction metric, the stoichoimetric factor (SF), has been decribed which allows to take into account reactions run under nonstoichoimetric conditions. Based on four competing factors (reaction yield, atom economy, stoichoimetric factor and a factor accounting for reaction and postreaction solvent and/or catalyst recovery) a general algorithm for reaction mass efficiency has been proposed. [20] This has been followed by the introduction of minimum atom economy (AE)_min_ and maximum environmental impact factor *E*_max_ that have been applied to over 400 named reactions. [21]

As can be seen from the discussion above, the search and implementation of the appropriate metrics for evaluation of the quality of a chemical process can be complex, time-consuming, not straightforward (unclear definitions) or too focused on one topic (waste, safety, etc.). In particular, the lack of transparency of the life cycle analysis, the lack of objectivity in assigning the Q value for a reagent or the unclear definition of "non-benign" for the calculation of effective mass yield, can be noted.

To our knowledge, no tool for evaluation of chemical reaction conditions on laboratory scale has been developed. Herewith, we propose a unified algorithm, called EcoScale, to help select an acceptable organic preparation.

## Design of the EcoScale

### Starting principles

A basic requirement for the design of the EcoScale is transparency and user-friendliness. At the same time, it needs to cover the whole range of organic chemistry conditions and techniques. To combine all these goals, the following approach is used.

First, the tool uses a scale from 0 to 100 with 0 representing a totally failed reaction (0% yield) and 100 representing the ideal reaction which is defined as follows: ***Compound A (substrate) undergoes a reaction with (or in the presence of) inexpensive compound(s) B to give the desired compound C in 100% yield at room temperature with a minimal risk for the operator and a minimal impact for the environment***.

Secondly, 6 general parameters which influence the quality of reaction conditions are analyzed (Table 1). Within each of these parameters, individual penalty points of various relative weights are assigned that take into account all possible situations when setting up an organic chemistry experiment. The penalty points are cumulative for all components of the preparation. In order to simplify the EcoScale design, the usual differentiation between solvents (usually present in > 10 equiv.), reagents, auxiliary or co-reagents and catalysts (usually present in < 0.1 equiv) is not made.

**Table 1:** The penalty points to calculate the EcoScale

Parameter	Penalty points

1. Yield	(100 – %yield)/2
2. Price of reaction components (to obtain 10 mmol of end product)	
Inexpensive (< $10)	0
Expensive (> $10 and < $50)	3
Very expensive (> $50)	5
3. Safety^a^	
N (dangerous for environment)	5
T (toxic)	5
F (highly flammable)	5
E (explosive)	10
F+ (extremely flammable)	10
T+ (extremely toxic)	10
4. Technical setup	
Common setup	0
Instruments for controlled addition of chemicals^b^	1
Unconventional activation technique^c^	2
Pressure equipment, > 1 atm^d^	3
Any additional special glassware	1
(Inert) gas atmosphere	1
Glove box	3
5. Temperature/time	
Room temperature, < 1 h	0
Room temperature, < 24 h	1
Heating, < 1 h	2
Heating, > 1 h	3
Cooling to 0°C	4
Cooling, < 0°C	5
6. Workup and purification	
None	0
Cooling to room temperature	0
Adding solvent	0
Simple filtration	0
Removal of solvent with bp < 150°C	0
Crystallization and filtration	1
Removal of solvent with bp > 150°C	2
Solid phase extraction	2
Distillation	3
Sublimation	3
Liquid-liquid extraction^e^	3
Classical chromatography	10

^a^Based on the hazard warning symbols. ^b^ Dropping funnel, syringe pump, gas pressure regulator, etc. ^c^ Microwave irradiation, ultrasound or photochemical activation, etc. ^d^scCO_2_, high pressure hydrogenation equipment, etc. ^e^If applicable, the process includes drying of solvent with desiccant and filtration of desiccant.

### Calculation of the EcoScale

An ideal reaction has the EcoScale value of 100. The EcoScale score for a particular preparation of the product in a high purity state (> 98%) is calculated by lowering the maximum value of 100 by any applicable penalty points.

**EcoScale = 100 - sum of individual penalties**

## Discussion

Although the choice of these 6 parameters will likely reach consensus among organic chemists, their relative weight and the assignments of the actual value of the penalties can raise a discussion. Specifically, it must be stressed that the relative weights of these parameters in the decision process fundamentally differ when the scale of the reaction is considered. Basically, the focus shifts away from the overall time and convenience on a laboratory scale, to the overall cost in industry when all regulatory restrictions are considered. In particular, no restrictions for using specific reagents/solvents exist on a laboratory scale, but high yield reactions can still be banned for larger scale production; for example, by using a highly flammable or toxic solvent or an expensive reagent. Similarly, reactions at room temperature are far more important on an industrial scale as no energy is needed for heating or cooling. Also, the waste issue is of a minor importance at a laboratory scale but can take up a significant cost of a production process. Therefore, it is important to stress that this EcoScale is specifically designed for laboratory scale conditions.

Even with the scale issue in mind, each weight of the parameters and each (relative) value of the penalty points are often only based on experience and intuition and not on "exact science". The subjective basis of these values in EcoScale is explained in more detail below. In particular, the subjective assignment of particular weights to various penalty points can easily be modified, as some chemists may disagree with the proposed relative assignments. The EcoScale is designed to be a flexible tool.

### 1. Yield

The yield is one of the most important factors. Indirectly, this parameter includes selectivity issues, as the quality of a reaction increases with increasing the functional group compatibility. An independent selectivity parameter would make the analysis highly complicated. A high yield guarantees an optimal use of resources and usually results in an easy workup procedure as side-products are limited. The question remains which yield to take, before or after purification of the product? Theoretically, the pre-purification yield is the best but is not practical to implement. First, this yield is often not determined (and not mentioned in the literature). In addition, the value of reaction conditions from which the end product cannot efficiently be purified is questionable. Therefore, points are calculated for the isolated yield.

The EcoScale analysis can also be applied to the evaluation of non-racemic synthesis. In this case, only the chemical yield of the targeted enantiomer is considered. The use of efficient chiral auxilaries can significantly raise the EcoScale (higher yield of enantiomer), but the final score is strongly influenced by their amount, availability and safety profile.

### 2. Price of reaction components

Every reaction component is taken into account, and the penalties are cumulative. The categorization of the reaction components as "inexpensive/readily available" is subjective. We define a reaction component as inexpensive if the cost to use it to synthesize the end product on a 10 mmol scale does not exceed US$10 and very expensive when its price is over US$50. We realize that by using this criterion, the EcoScale is time dependent. A reaction component that is not commercially available today might appear in the catalogues next year and, as such, will rank higher on the EcoScale in the future. This is only fair because the evaluation process is also time dependent: we can refrain from using certain reagents today because we would need to synthesize them, but might use them in the future when they become commercially available.

Reaction components present in over 10 equivalents in the reaction mixture are usually solvents and often are inexpensive. However, common solvents used under strictly anhydrous and/or high-dilution (large volume) conditions should be re-evaluated as expensive components. The use of an expensive solvent (e.g. ionic liquid) does not necessarily mean a lower score on the EcoScale, as a higher yield, a better safety profile or easier workup can favourably balance the score. In addition, two special cases can be noted. In a solvent-free reaction and when the solvent is used as the reagent, there is one reaction component less for which no extra penalties are deducted. It must be noted that the physicochemical characteristics are not taken into account here: solvents with a boiling point higher than 150°C (DMF, DMSO, diglyme, DMA, HMPTA etc.) and lower than 25°C (e.g. scCO_2_) are penalized but in a different category (workup and technical setup, respectively).

Similarly, the price of reaction components present in a small amount (usually catalysts) is determined by the mol% needed. An expensive but efficient catalyst (e.g. with high turnover number, low mol% needed) can qualify as an inexpensive component. The same catalyst can have a price penalty if used in another reaction in 10 mol% ratio. It must be stressed that the real benefits of using catalysts usually are reflected in higher selectivity (yield) and lower energy requirements, which are accounted for in other parts of the EcoScale.

### 3. Safety

Safety is of paramount importance when carrying out organic chemistry experiments. Working with chemicals is never without a risk, and it is necessary to fully understand any potential hazard. Organic compounds can be carcinogenic, mutagenic, teratogenic, corrosive, lachrymatic, highly flammable or explosive, among other things. In addition, the hazard can increase over time, and photooxidation of ether to generate explosive peroxides is a good example. It must also be emphasised that it takes a long time before the safety profiles of new products are fully characterized. Finally, one should never forget that the combination of certain individual compounds can create a hazardous situation (e.g. exothermic reaction between acids and bases).

For assessing these hazards, a wide variety of information is readily available, such as the health and safety information in Risk/Safety phrases, the Material Safety Data Sheets, and the hazard warning symbols on the containers. In order to avoid a complex calculation, the hazard warning symbols are taken as a reference. In particular, each reaction component labelled with T+ (extremely toxic), F+ (extremely flammable) or E (explosive) is penalized with 10 points while reaction components labelled with T (toxic), F (highly flammable) or N (dangerous to the environment) are given 5 penalty points. [22] As can be seen from Table 1, the use of unsafe compounds can downgrade the overall quality of synthesis to the greatest extent in comparison to other entries.

### 4. Technical setup

A simple setup consisting of a regular flask, reflux condenser, and stirrer receives no penalty points. Any extras including special glassware, equipment for controlled addition of chemicals, pressurized vessels, the application of unconventional techniques such as microwave irradiation, ultrasound or photochemistry, and the need for an inert atmosphere, especially in a glove box, downgrade the overall quality of the synthesis.

### 5. Temperature/time

The reaction temperature and time are closely related. In an ideal situation, a reaction proceeds rapidly at room temperature. However, heating is often required to accomplish synthesis in an acceptable period of time. On the other hand, cooling is more difficult than heating. Above room temperature, the heating range is continuous while for cooling in a conventional way (without the use of a cryostat) only fixed temperatures (e.g. 0°C for ice bath or -78°C for acetone/sCO_2_) are available, and great care must be taken sometimes to avoid moisture in order to produce reproducible results. These features are reflected in the relative penalty points. The penalties are cumulative; if heating and cooling are required during the reaction, both must be accounted for.

### 6. Workup and purification

The workup and purification of the end product can be a tedious process. In order to avoid a complex calculation (e.g. when taking into account all used chemicals), the factor "a period of time to obtain the end product in a purity of over 98%" is taken as the main criterion in assigning the points. As it makes no sense to use a chronometer in a laboratory workup procedure, standard purification techniques are ranked according to their execution time (and convenience). Every workup step is taken into account in assigning the penalty points.

#### Ranking of reaction conditions

The reaction conditions used in the preparation of a high purity (> 98%) product is ranked on a scale from 0 to 100 using the following scores: > 75, excellent; > 50, acceptable; and < 50, inadequate.

#### Examples of calculations

The EcoScale evaluations of four important synthetic transformations taken from the recent literature are shown below. Workup involves manipulations in the given order.

The sum of all penalty points is 36 (Table 2), which gives total score of 64 on the EcoScale (an acceptable synthesis).

**Scheme 1:**
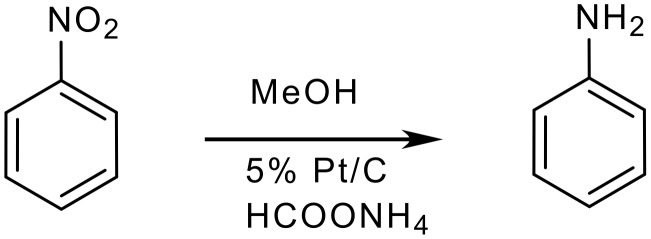
Reduction of nitrobenzene to aniline [23]

**Table 2:** The penalty points for example 1

# 1–6 from Table 1	Penalty

1 Yield: 90 %	5
2 5% Pt/C, 0.3 g	3
3 Nitrobenzene (T, N)	10
MeOH (T, F)	10
5% Pt/C (F)	5
4 Common glassware, stirring	0
5 Room temperature, 1 h	0
6 Filtration of the catalyst	0
Removal of MeOH	0
Addition of CHCl_3_	0
Washing with aq. NaCl	3
Removal of CHCl_3_	0

Penalty points total:	36

The sum of all penalty points is 22 (Table 3), which gives total score of 78 on the EcoScale (an excellent synthesis).

**Scheme 2:**
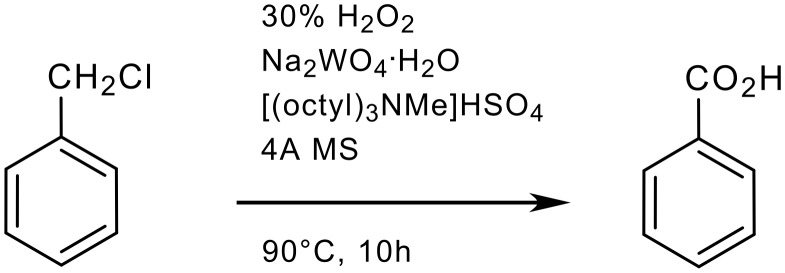
Oxidation of benzyl chloride to benzoic acid [24]

**Table 3:** The penalty points for example 2

# 1–6 from Table 1	Penalty

1 Yield: 87%	6
2 H_2_O_2_ (30%, 4.1 mL, 36 mmol)	0
Na_2_WO_4_·2H_2_O (66 mg, 0.2 mmol)	0
[(Octyl)_3_NMe]HSO_4_ (93 mg, 0.2 mmol)	0
Molecular sieves 4Å (100 mg)	0
3 Benzyl chloride (T)	5
4 Dropwise addition of H_2_O_2_	1
5 90°C, 10 h	3
6 Extraction with AcOEt (3 × 10 mL)	3
Washing with aq. Na_2_S_2_O_4_	3
Drying over MgSO_4_	0
Removal of AcOEt	0
Crystallization from hexanes	1

Penalty points total:	22

The sum of all penalty points is 47 (Table 4), which gives total score of 53 on the EcoScale (an acceptable synthesis).

**Scheme 3:**
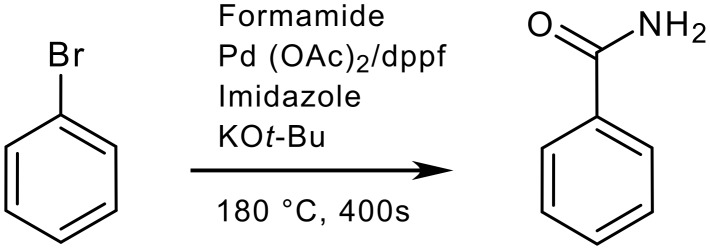
Synthesis of benzamide [25]

**Table 4:** The penalty points for example 3

# 1–6 from Table 1	Penalty

1 Yield: 83%	9
2 Formamide (1 mL)	0
Pd(OAc)_2_ (0.038 mmol, 8.5 mg)	0
dppf (0.038 mmol, 21.1 mg)	0
Imidazole (0.75 mmol. 51.1 mg)	0
KOBu-*t* (1.13 mmol, 126 mg)	0
3 Bromobenzene (N)	5
Formamide (T)	5
KOBu-*t* (F)	5
dppf (T)	5
4 Microwave activation	2
Nitrogen atmosphere	1
5 180°C, 400 s	2
6 Cooling	0
Dilution with EtOAc	0
Washing with water and brine	3
Drying over potassium carbonate	0
Removal of EtOAc	0
Silica gel chromatography	10

Penalty points total:	47

In the introduction to the article, [25] the authors claim that this procedure for preparing primary amides starting from aryl halides is better than another procedure which uses hexamethyldisilazane (HMDS). [26] Therefore, the EcoScale for the latter procedure was also calculated to compare the two preparations.

The sum of all penalty points is 68 (Table 5), which gives total score of 32 on the EcoScale (an inadequate synthesis).

**Scheme 4:**
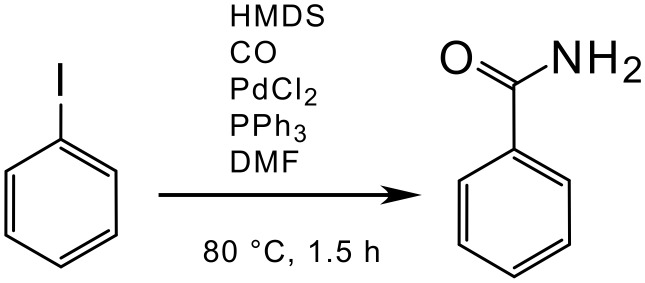
Synthesis of benzamide using HMDS [26]

**Table 5:** The penalty points for example 4

# 1–6 from Table 1	Penalty

1 Yield: 76%	12
2 HMDS (4 equiv.)	0
CO (in excess)	0
PdCl_2_ (0.03 equiv.)	0
PPh_3_ (0.06 equiv.)	0
DMF	0
3 CO (T, F+)	15
HMDS (F)	5
DMF (T)	5
PPh_3_ (N)	5
Bromobenzene (N)	5
4 Controlled addition	1
CO atmosphere	1
5 80°C, 1.5 h	3
6 Cooling	0
Addition MeOH	0
Addition 2N H_2_SO_4_	0
Extraction with AcOEt	3
Washing with aq.NaHCO_3_ and brine	3
Silica gel chromatography	10

Penalty points total:	68

This procedure receives a significantly lower score than the previous example largely due to its safety profile and the more tedious workup. By using EcoScale, the two analyses (#3 and #4) illustrate a rapid selection of the better preparation (#3).

## Conclusion

In general, the EcoScale favours high-yielding, low-cost and safe reaction conditions and an easy purification. The analysis (1) is straightforward (it takes into account all important parameters), (2) is transparent (it is clear how the final score is obtained), (3) is fast (it can be calculated in less than 5 min) [27], (4) does not take a general standpoint but takes into account advantages and disadvantages of specific methodologies or auxiliary reagents, (5) offers a general overview of the reaction conditions, and the areas for improvement are clearly indicated. In this way, it can be used as a convenient tool in education (students learn to analyze a reaction protocol), and is valuable in research as an effective way to compare different sets of preparations of the same product.
